# Assessing the utility of a novel cortical marker of delay discounting (C-DD) in two independent samples of early adolescents: Links with externalizing pathology

**DOI:** 10.1371/journal.pone.0291868

**Published:** 2023-09-27

**Authors:** Nadia Bounoua, Leah D. Church, Melanie A. Matyi, Jeremy Rudoler, Kaleigh Wieand, Jeffrey M. Spielberg

**Affiliations:** 1 Department of Psychology, University of Maryland, College Park, Maryland, United States of America; 2 Department of Psychological & Brain Sciences, University of Delaware, Newark, Delaware, United States of America; Kyoto University Graduate School of Informatics: Kyoto Daigaku Daigakuin Johogaku Kenkyuka, JAPAN

## Abstract

Delay discounting is a well-established risk factor for risky behaviors and the development of externalizing spectrum disorders. Building upon recent work that developed a novel cortical marker of delay discounting (C-DD) in adult samples, the objective of this study was to test whether the C-DD relates to delay discounting and subsequently externalizing pathology in adolescent samples. The current study used two samples: 9992 early adolescents participating in the ABCD study (M_age_ = 9.93 years old, 48.7% female), and 56 early adolescents recruited from the community (M_age_ = 12.27 years old, 55.4% female). Cortical thickness was estimated using the FreeSurfer standard pipeline, and the cortical marker of delay discounting (C-DD) was calculated based on procedures outlined by the initial validation study. All data are cross-sectional in nature. As expected, C-DD was positively related to delay discounting in the ABCD sample, even after accounting for age, biological sex, collection site and data quality indicators. Moreover, results showed that C-DD was discriminately associated with externalizing, but not internalizing, symptoms in both samples of young adolescents. Findings replicate those found in adult samples, suggestive that C-DD may be a useful neuroanatomical marker of youth delay discounting. Replication of findings in other samples will be needed to determine whether C-DD has translational relevance to understanding externalizing psychopathology in adolescent samples.

## Introduction

Adolescence has been well-established as a sensitive period marked by increased engagement in risk-taking behaviors [1, 2]. Despite this recognition, the mechanisms driving this increase in risk-taking remain largely unknown, rendering existing prevention efforts largely ineffective [3]. This is particularly lamentable given that engagement in risky behaviors continues to be the leading cause of preventable injuries and deaths among children and adolescents in the United States [4]. Thus, more research is critically needed to identify risk factors that can be used to understand risky behaviors and associated mental health problems early in adolescence. In particular, there has been a push for incorporating a neuroscience approach to understanding adolescent risk-taking [3, 5], and this is one of the fundamental objectives of the ABCD Consortium [6].

Accumulating evidence suggests that delay discounting, or the extent to which individuals tend to prefer smaller, sooner rewards over a larger, later rewards, is a robust behavioral predictor of a myriad of risky behaviors. The majority of existing work on delay discounting has centered on its associations with externalizing psychopathologies, given that these disorders are characterized by poor self-regulation, alterations in reward-related processes, and impulsive decision-making [7, 8]. For example, extant research to date has linked delay discounting to addiction and substance-use behaviors [9–15]. Indeed, meta-analytic work shows that delay discounting is associated with addiction [16], and research with adolescent samples have consistently demonstrated higher delay discounting rates among substance-using adolescents [17–19]. Other studies have extended these findings by demonstrating links between delay discounting and symptoms across the externalizing spectrum disorders, including aggression, rule-breaking, and risky sexual behaviors [20–23].

Given its relevance to risky behavior, research has sought to examine the neurobiological underpinnings of delay discounting. Previous work suggests that delay discounting is associated with various neural structures implicated in reward valuation (e.g., ventromedial PFC), cognitive control (e.g., lateral PFC), and prospection and future planning (e.g., middle temporal lobe; medial PFC) [6, 24–30]. Furthermore, neuroimaging studies consistently link steeper delay discounting (greater preference for immediate rewards) to less structural integrity across the cortex (i.e., thinner cortex and/or smaller volume) [31–33]. Given accumulating evidence that the valuation of future rewards is instantiated in multiple cortical sites, research attempting to index the neural variation linked to delay discounting must incorporate a multivariate approach.

Sadeh and colleagues [34] developed a novel cortical assay of delay discounting (C-DD) in adults that fits the criteria described above. Specifically, the C-DD marker was established by individually regressing the average thickness of each cortical parcellation in both hemispheres (derived from the FreeSurfer Destrieux Atlas) [35] on performance on a well-validated delay discounting task in a sample of over 1,000 adults participating in the Human Connectome Project [36]. The decision to restrict their analyses to cortical thickness, as opposed to other morphometric measures (e.g., subcortical volume), was based on (i) research demonstrating that total cortical (but not total subcortical) gray matter volume was inversely related to delay discounting [32] and (ii) cortical thickness is a reliable and stable neurobiological marker that can be readily assessed using routinely-collected T1 anatomical scans [37, 38]. To compute an individual’s C-DD score, each of the resultant 148 standardized beta (regression) weights were multiplied by the thickness of the relevant parcellation and the results summed to create a total C-DD score for that individual, with higher C-DD scores indicating thinner cortex, given that the majority of regression weights were negative. In two independent samples of adults, the association between C-DD and delay discounting was replicated. Furthermore, C-DD was associated with known correlates of delay discounting, including cannabis use and externalizing pathology. Importantly, these relationships with C-DD remained significant even with a behavioral measure of delay discounting included in the model, indicating that this cortical assay captures relevant variance beyond the behavioral task upon which it was based. Based on these preliminary data, the authors conclude that C-DD may represent a neurobiological marker of impulsive choice and externalizing pathology in adult samples [34].

### Current study

Given associations between delay discounting and later behavioral outcomes, a neurobiological marker like the C-DD that assays the tendency to engage in impulsive decision-making has the potential to be an indicator of adolescent’s vulnerability for engagement in risky behavior and related pathology. However, given that adolescence is a period of rapid neurodevelopmental changes, particularly in regions associated with cognitive control, reward valuation and emotional process [2, 25], it is unclear whether the adult-derived C-DD would be an effective indicator in this period. Thus, the objective of the present study was to test associations between the C-DD marker and externalizing spectrum psychopathology in two independent samples of early adolescents: (i) a large sample of adolescents from the Adolescent Brain Cognitive Development^SM^ (ABCD) Study and (ii) a smaller sample of adolescents recruited from the community. Specifically, we used the betas derived in the original adult C-DD study to create a C-DD score for each participant (using the method described above) in both adolescent samples (see S1 Appendix for C-DD weights and further details on calculation). We opted to use this approach rather than deriving regression weights specific to the adolescent sample, because our focal objective in this study was to test whether the adult-derived C-DD marker was also a useful indicator of similar psychological processes in adolescent samples. Thus, we were able to test the C-DD’s performance in two adolescent samples that were independent of the (adult) sample from which the regression weights were derived. One potential limitation of this approach is that there may be neural relations unique to adolescent delay discounting that are not captured in the adult-derived C-DD marker. Of course, it is possible to employ the same validation method used in the original C-DD paper in the adolescent sample to identify potential adolescent-specific regions/weights related to delay discounting. However, given vast changes in neurodevelopment during early adolescence, one limitation of an adolescent-derived C-DD marker would be that its utility may be limited to adolescence, or even early adolescence, in particular. Instead, the adult C-DD marker could capture variance that transcends a particular developmental period, and it was the purpose of this study to assess whether this was true. Given these considerations, we opted to test the utility of the adult-derived C-DD marker in adolescent samples to test its utility in adolescent samples and maximize compatibility across studies.

## Materials and methods

### Participants

Sample demographics for both studies can be found in Table 1.

**Table 1:** Sample demographics.

			Sample 1 n (%)	Sample 2 n (%)
Age (in years); M/SD			9.93 (0.63)	12.27 (0.94)
Sex				
Male			4870 (51.3)	25 (44.6)
Female			5122 (48.7)	31 (55.4)
Race				
Black/African American			1618 (16.2)	11 (19.6)
White			7409 (74.1)	37 (66.1)
Asian			466 (4.6)	4 (7.4)
American Indian/Native Alaskan			223 (2.2)	1 (1.8)
Native Hawaiian/Pacific Islander			47 (0.5)	0 (0)
Ethnicity				
Hispanic			1726 (17.3)	4 (7.1)
Non-Hispanic			8205 (82.1)	43 (76.8)
Family Income (past 12 months)				
< $5,000			1656 (16.6)	2 (3.6)
$ 5,000- $9,999			829 (8.3)	0 (0)
$10,000-$15,999			517 (5.2)	0 (0)
$16,000-$24.999			797 (8.0)	2 (3.6)
$25,000-$34.999			960 (9.6)	4 (7.1)
$35,000-$49.999			1182 (11.8)	4 (7.1)
$50,000-$74.999			1446 (14.5)	11 (19.6)
$75,000-$99,999			800 (8.0)	7 12.5)
> $100,000			902 (9.0)	14 (25)
Psychopathology	Mean/SD (Range)	Mean/SD (Range)		
Externalizing Symptoms	4.38/5.80 (0–49)	6.98/5.51 (0–20)		
Internalizing Symptoms	5.03/5.54 (0–51)	8.09/7.00 (0–31)		

#### Study 1

We examined data from 9,992 early adolescents whose FreeSurfer cortical parcellations passed the ABCD^SM^ quality assurance checks (https://abcdstudy.org/scientists/data-sharing/). ABCD^SM^ is a large longitudinal study that recruited children across 21 research sites in the United States. More details of study design and MRI preprocessing can be found at the ABCD website (https://abcdstudy.org/scientists/protocols/) and is described elsewhere [39, 40]. Informed consent was provided prior to data collection. At the time of assessment, youth ranged from 8.91–11.08 years old (M/SD_age_ = 9.93/0.63), with approximately half of the sample (48.7%) reporting female biological sex.

#### Study 2

A community sample of 56 early adolescents between the ages 11–14 years old (M/SD_age_ = 12.27/0.94; 55.4% female) were recruited as part of a larger study examining the neural development related to emotion regulation and psychopathology between 2019 and 2021. Adolescents and their families were eligible to participate in the study if they were between the ages of 11–14 and fluent in English. Adolescents did not have to meet a clinical diagnosis to be included in the study. Exclusion criteria included: current youth or parental psychosis, history of head injury with loss of consciousness for over 30 minutes or lasting effects, serious medical or neurological condition, current pregnancy, or MRI contraindications. All participants who had complete MRI data were included. Research staff had access to information that could identify individual participants during or after data collection. The University of Delaware, Institutional Review Board approved this research (1464167). Written consent and assent were obtained prior to data collection procedures.

### Materials

An overview of the study variables and their measurement are described below and can be found in Table 2.

**Table 2:** Overview of measurement across samples.

	Sample 1	Sample 2
Cortical Delay Discounting Marker	X	X
Delay Discounting	X	
CBCL–Externalizing		
CBCL–Internalizing	X	X

*Note*. CBCL = Child Behavior Checklist [50]

#### Cortical thickness. *Studies 1 & 2*

Thickness of the cortical mantle was estimated using FreeSurfer’s (v6) standard morphometric pipeline [41–43]. Information regarding the MRI preprocessing pipeline of the ABCD dataset (Sample 1) can be found on the ABCD website (https://abcdstudy.org/scientists/protocols/) and is described elsewhere [6, 39, 44, 45]. For Sample 2, T1 and T2 images were visually inspected and at least two trained raters examined the data for errors, including the inclusion of dura or skull after brain extraction or errors in the pial or white matter surfaces. Cortical thickness was calculated for each parcellation derived from the FreeSurfer Destrieux Atlas, which parcellates the cortex into 74 neuroanatomically-distinct structures in each hemisphere [35, 46].

*Cortical Delay Discounting (C-DD) marker (Studies 1 & 2)*. We calculated a cortical delay discounting (C-DD) using the same standardized weights reported in Sadeh et al. [34]. Specifically, we computed a total C-DD score by weighting the z-scored cortical thickness for of the 148 FreeSurfer Destrieux Atlas parcellations [35] by its corresponding standardized beta coefficient derived from Sadeh et al. [34] and summing the resulting values (see S1 Appendix). This procedure was used in both adolescent samples in the present study. The motivation to use the adult-derived betas (as opposed to obtaining adolescent-derived betas) was to maximize compatibility across studies and test whether the C-DD obtained using an adult sample has utility in adolescent samples.

#### Delay discounting (*Study 1 only)*

Delay discounting was measured using the 5-trial adjusting delay discounting task [47]. Full details about this publicly-available task can be found at https://www.millisecond.com. Briefly, participants were asked to make a choice between a delayed ($100) and immediate amount, which is adjusted based on participant’s choice on the previous trial. Seven delays ("6 hours", "1 day", "1 week", "1 month", "3 months", "1 year", "5 years") were tested to determine seven indifference points. The “indifference point” is the point where a small- immediate reward is equal in value to a delayed but larger reward. These indifference points typically form a hyperbolic curve [48] whose steepness is defined by a discounting constant *k* [12], which was estimated by Mazur’s hyperbolic equation [49]. The estimated *k*-values were natural log-transformed to account for non-normality of discount rates, with larger ln*k* values indicating a greater degree of discounting future rewards.

#### Psychopathology (*Studies 1 & 2)*

The 112 item Child Behavior Checklist for 6-18-year-old children—Parent Version (CBCL/6-18) [50] was used to assess child psychopathology. The CBCL is a well-validated and widely-used measure of child psychopathology [51–53]. Parents were asked to indicate the response option that best described their child on a sale from 0 ("not true"), 1 (“somewhat/sometimes true”) or 2 ("very true") and responses were elicited from one parent per child. Consistent with the CBCL scoring instructions, an externalizing composite score was created by summing the 35 items from the rule-breaking (e.g., “Drinks alcohol without parents’ approval.”) and aggressive behavior (e.g., “Gets in many fights.”) subscales. An internalizing composite score was composed by summing the 31 items from the anxious/depressed (e.g., “Nervous, high-strung, or tense”), withdrawn/depressed (e.g., “Unhappy, sad, or depressed”), and somatic complaints (e.g., “Has stomach aches without a known medical cause.”) subscales. Although previous research has shown that the CBCL shows strong diagnostic accuracy [53–55], this measure is not a diagnostic tool. In line with this dimensional approach, higher composite scores indicate greater symptom severity.

### Data analysis

First, we conducted preliminary analyses to examine bivariate associations between C-DD and criterion variables of interest (e.g., delay discounting, impulsivity, psychopathology symptoms). Next, we conducted a partial correlation to test whether C-DD was associated with behavioral delay discounting, after accounting for age, biological sex, and Freesurfer quality control motion parameters in Sample 1. Finally, Generalized Linear Modeling was used to test whether C-DD was associated with youth psychopathology in both samples. This analytics approach was chosen to accommodate the non-normal distributions of key study variables (see S1 and S2 Figs). Specifically, we specified a gamma distribution with log link model, with bootstrapping (1000 samples) and 95% confidence intervals. Age and biological sex were included as covariates of no interest in all multivariate analyses. All analyses were conducted in SPSS v.28.0 [56]. It should be noted that all external correlates of C-DD were measured at the same time point, and thus our findings reflect concurrent associations. A portion of these data used in the preparation of this article (Study 1) were obtained from the Adolescent Brain Cognitive DevelopmentSM (ABCD) Study (https://abcdstudy.org), held in the NIMH Data Archive (NDA). All relevant data for Study 2 are within the manuscript and its Supporting Information files.

## Results and discussion

### Sample 1

Preliminary analyses revealed that C-DD scores were positively associated with age (*r* = .12, *p* < .001), such that older youth had higher C-DD scores than younger adolescents. Significant sex differences emerged in relation to C-DD scores (*F*_(1,9990)_ = 11.74, *p* < .001), such that boys (*M/SD* = -.186/1.77) had larger C-DD scores than girls (*M* = -.185/1.78). Internalizing and externalizing symptoms were moderately correlated (*r* = 0.59, *p* < 0.001).

#### Delay discounting

As expected, C-DD was positively correlated with ln*k*-values at a bivariate level (*r* = .04, *p* < .001), indicating that higher values on this cortical assay correspond to greater impulsive choice. Importantly, this relationship remained significant when controlling for age, biological sex, MRI quality control measures, and data collection site (β = .04, *p* < .001).

#### Psychopathology

Consistent with past work, C-DD was associated with externalizing symptoms at a bivariate level (*r* = .02, *p* = .02), such that greater C-DD scores were associated with more externalizing symptoms. To test the incremental validity of C-DD over behavioral measures of impulsivity, We then conducted a generalized linear model to test whether C-DD continued to be associated with externalizing symptoms, after controlling for behavioral delay discounting (ln*K*), age, sex, and (see Table 3). Consistent with past work, C-DD remained a significant predictor of externalizing pathology above and beyond behavioral delay discounting (ln*k*-values).

**Table 3:** C-DD is incrementally associated with externalizing pathology among early adolescents, above and beyond behavioral delay discounting (Sample 1).

	B	SE	*p*	*95% CI*
Biological sex	-.031	.003	< .001	-.037, -.025
Age	-.001	.0002	.007	-.001, -.0001
ln-*K*	.001	.001	.062	-.00001, .002
C-DD	.002	.001	.043	.00005, .004

*Note*. Results from Generalized Linear model with gamma distribution and log link. Bootstrapping with 1,000 samples and 95% confidence intervals (CI). Biological sex is coded as 1 (male), 2 (female). ln-*K =* natural log-transformed *k*-value (behavioral delay discounting). C-DD = cortical delay discounting marker. Pearson chi-square = 193.95_(9290)_, *p* = .021.

As expected, C-DD scores were not associated with internalizing symptoms, after accounting for age, sex, and ln*K [*(*B(SE)* = -.0002(.001), *p* = .80)], suggesting that C-DD is discriminately associated with externalizing spectrum pathology.

### Sample 2

C-DD was not associated with age (*r* = 0.11, *p* = 0.41) or biological sex (*F*_(1,54)_ = 0.18, *p =* 0.67) in this sample, likely due to the smaller sample size. Internalizing and externalizing symptoms were moderately correlated (r = 0.58, p < 0.001). Delay discounting behavior was not assessed in this sample, and thus relationships with ln*k* could not be examined.

#### Psychopathology

**Fig 1:**
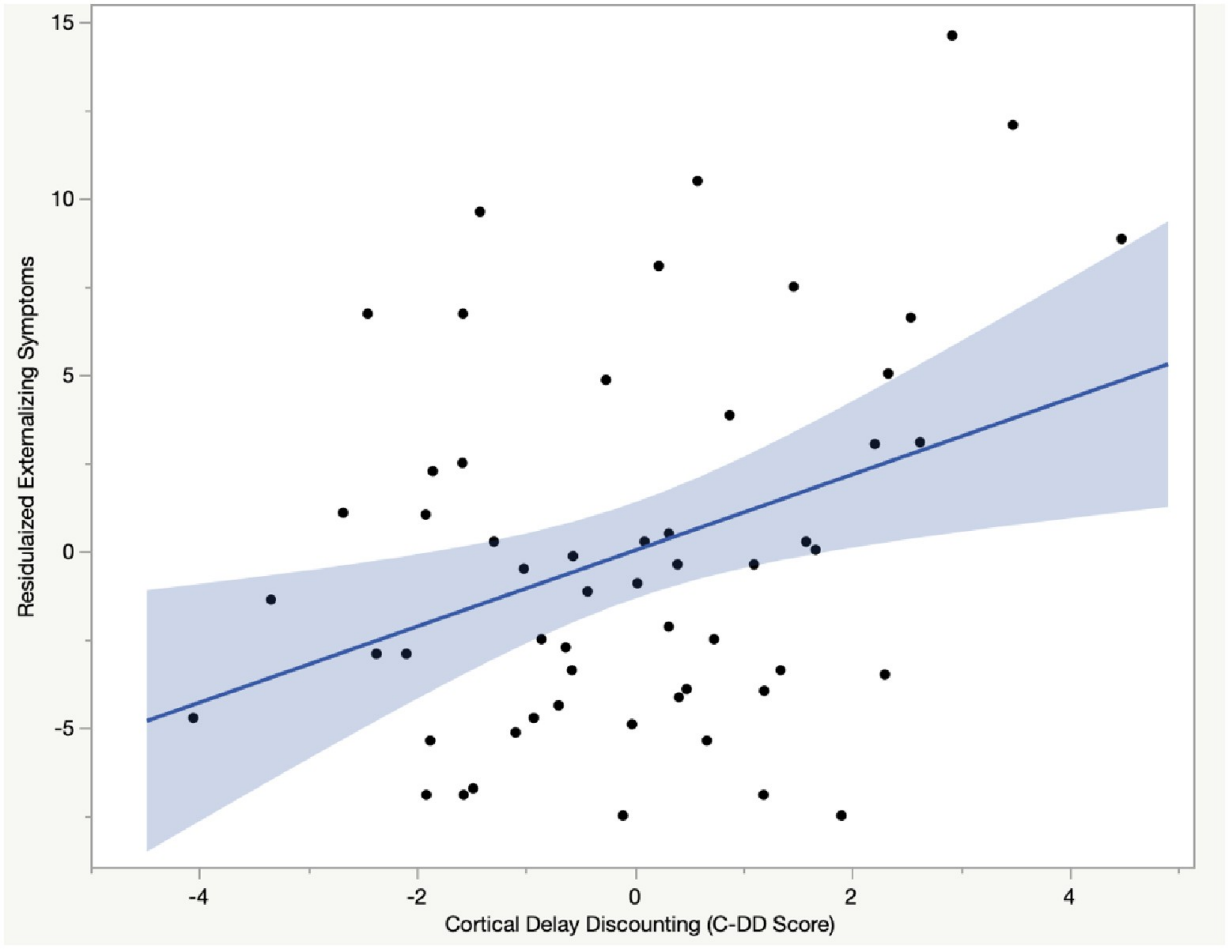
C-DD is positively associated with externalizing symptoms in Sample 2 (after accounting for covariates).

Consistent with past work and the findings in the ABCD sample, C-DD was positively associated with externalizing symptoms (*r* = 0.32, *p =* 0.016), indicating that greater C-DD scores were associated with more rule-breaking and aggressive behaviors (see Fig 1). Results of a hierarchical linear regression revealed that the positive association between C-DD and externalizing symptoms remained significant after including age and biological sex in the model (see Table 4). As expected, C-DD scores were not associated with internalizing symptoms, after accounting for age and biological sex [(B(SE) = .017(.013), p = .163)], suggesting that C-DD is discriminately associated with externalizing spectrum pathology.

**Table 4:** C-DD is associated with externalizing pathology among early adolescents (Sample 2).

	B	SE	*p*	*95% CI*
Biological sex	-.068	.033	.040	-.141, .014
Age	-.026	.018	.150	-.065, .014
C-DD	.013	.003	.003	.006, .044

*Note*. Results from Generalized Linear model with gamma distribution and log link. Bootstrapping with 1,000 samples and 95% confidence intervals (CI). Biological sex is coded as 1 (male), 2 (female). C-DD = cortical delay discounting marker. Pearson chi-square = 0.770_(52)_, *p* = .015.

## Conclusions

The objective of the present study was to investigate the utility of a novel whole-cortex marker of delay discounting for understanding impulsive decision making and externalizing pathology among early adolescents. Consistent with findings from the initial validation study in adults [34], we found that the cortical delay discounting marker (C-DD) was associated with delay discounting rates in the ABCD dataset. Moreover, C-DD was associated with greater externalizing, but not internalizing, psychopathology. Importantly, C-DD showed incremental predictive validity above and beyond behavioral delay discounting measures. Evidence of cross-validation was also identified, such that C-DD was associated with externalizing, but not internalizing symptoms in a smaller community sample of early adolescents. Taken together, findings of the current study provide initial support for the utility of using the C-DD as a potential neuroanatomical assay of *adolescent* impulsive decision-making that may have translational relevance to externalizing pathology and risk-taking in this sensitive population.

### Utility of a cortical marker of adolescent delay discounting

Adolescence has been consistently characterized as a sensitive period for impulsive decision-making [57, 58], placing adolescents at increased risk for poor outcomes. Thus, it is important to understand correlates of impulsive decision-making during this sensitive period across multiple levels of analysis. A wealth of literature has identified links between delay discounting and a range of pathological behaviors, and neuroimaging research has implicated numerous neural regions across the cortex associated with impulsive decision-making (see review above). A whole-cortex biomarker that indexes individual differences in impulsive decision-making has potential to identify youth who may be at a greater likelihood of engaging in externalizing behaviors. Consistent with findings in adult samples [34], we found that C-DD was significantly associated with a greater tendency to prefer smaller, sooner rewards in a large sample of early adolescents.

It should be noted that the size of this effect would be considered small (*Pearson’s r* = 0.04) using traditional effect size conventions [59]. New conventions have been proposed for interpreting effect sizes when using large sample sizes, such as the ABCD database [60]. In this framework, the observed effect size would fall within the “average” range and would be considered meaningful given the sample size. Further, the effect size is consistent with effect sizes reported by other studies using the ABCD dataset [61–63]. At the same time, the effect size leaves the true predictive power of the C-DD unclear, and thus future work is needed to determine the potential research and clinical utility of the C-DD.

Nonetheless, our findings provide preliminary support for the validity of C-DD as a cortical assay of delay discounting among early adolescents. Ultimately, the validation of the C-DD for adolescent populations opens avenues for future research interested in examining correlates of adolescent decision-making. Given that the C-DD can be calculated solely from T1 structural scans, which are collected in all MRI studies and are commonly collected in clinical settings, researchers can derive the C-DD metric in existing adolescent neuroimaging datasets that may not include behavioral measures of delay discounting. Further, current delay discounting paradigms vary in design and may conflate the tendency to engage in impulsive decision-making with individual differences in orientation to the future [5]. Given that C-DD is independent of these specific task parameters, it can be used to facilitate reproducible research on the correlates of adolescent delay discounting across multiple datasets. Accumulating empirical evidence demonstrating correlates of a neural marker of adolescent decision-making has the potential to advance the understanding of normative and disrupted neurodevelopment as it relates to real-world behaviors. To encourage this research, procedures for calculating the C-DD have been included in the S1 Appendix (see Supplementary Material).

### C-DD and adolescent externalizing pathology

Delay discounting is situated within the NIMH Research Domain Criteria [64], with recent work highlighting delay discounting as a particularly useful marker with transdiagnostic relevance [15]. The second aim of this study was to examine whether C-DD was associated with externalizing pathology. Using two independent sample, we found support for our hypothesis. As expected, findings showed that C-DD was selectively associated with externalizing symptoms, such as rule-breaking and aggressive behaviors, in both samples of adolescents. Evidence of discriminant validity was also observed in that C-DD was not associated with internalizing pathology (e.g., depression, anxiety). Although preliminary, these associations suggest that the C-DD may be used as a marker of impulsive decision-making that has clinical relevance to youth samples in studies that have T1 scans but did not collect behavioral or self-report measures of delay discounting. Furthermore, findings from Study 1 revealed that the C-DD had incremental validity in predicting externalizing pathology in the ABCD sample above and beyond a behavioral measure of delay discounting (lnK), indicating that C-DD explains unique variance in externalizing pathology not accounted for by a behavioral delay discounting paradigm alone. Thus, the C-DD may serve as a useful additional indicator, even in existing datasets that contain behavioral measures of delay discounting. Indeed, despite robust associations between behavioral delay discounting measures and risky behaviors, research shows that these paradigms do not tend to correlate with other self-reports of self-control or impulsivity [5, 65] pointing to the multifaceted nature of reward valuation and decision-making processes. Given the complexity of these processes, a multi-level approach that encompasses self-report, behavioral, and neuroanatomical measures of delay discounting may be necessary to elucidate associations between impulsive decision making and psychopathology. Given the relative stability of cortical thickness measures [66], one exciting potential is that the C-DD, along with other measures, could be used to identify at-risk youth prior to the engagement in risky behaviors.

### Strengths, limitations, & future directions

This study has several notable strengths, including the validation of C-DD using two samples of early adolescents, one of which was extremely large and the examination of convergent and divergent validity of C-DD. However, findings should be interpreted in light of study limitations. First, the samples in this study were predominately white and non-Hispanic/Latinx and recruited from the community. Thus, future research should seek to replicate these findings in other samples with more diverse samples across different settings, such as clinical samples. Second, the use of a multisite data collection study may allow for greater variability in experimental error [67]. Third, the original creation of C-DD consisted of only cortical thickness; however, research has also linked subcortical regions to impulsivity and decision-making processes [27, 68]. Thus, future research should examine how the interplay between C-DD and structural properties of subcortical regions relates to adolescent delay discounting and other impulsivity-related endophenotypes. Fourth, the current study relied solely on cross-sectional data, limiting our ability to make causal inferences of the associations between C-DD and adolescent externalizing pathology. Important areas for future research will be to assess the stability and predictive validity of the C-DD over the course of adolescence, and its ability to predict future psychopathology using longitudinal data.

Important areas for future research will be to assess the stability and predictive validity of the C-DD over the course of adolescence, and its ability to predict future psychopathology and other impulsive-based outcomes of interest. The C-DD was intentionally created as a whole-cortex marker, based on previous studies demonstrating that the tendency to discount future rewards (i.e., delay discounting) is not limited to specific cortical regions, but instead is associated with neural regions across the cortex [32]. One potential interpretation of these findings is that delay discounting is a complex phenomenon that likely involves several psychological processes that are instantiated in multiple places across the cortex. Future research will be needed to test for the specific mechanisms that link C-DD to endophenotypes like delay discounting and related-psychopathologies in order to further elucidate the driving forces behind the present findings. Moreover, given recent initiatives to apply transdiagnostic approaches to psychopathology [69], future work should also seek to examine the extent to which C-DD relates to dimensions and clusters of adolescent externalizing psychopathology using data-driven methods. This line of work would provide informative clarification on the specificity of C-DD to different dimensions of adolescent psychopathology. Similarly, future studies could test whether C-DD is associated with externalizing psychopathology at a diagnostic level using clinical interviews, as well as the impact of such psychopathology on adolescent functioning. Upon replication of these findings, the C-DD may also be used to inform etiological models of youth externalizing pathology and has the potential to be used in preventative measures by identifying early adolescents at greatest risk for developing impulse-control related pathology.

## Supporting information

S1 Appendix Calculation of C-DD originally cited by Sadeh et al (2023). (DOCX)

S1 Dataset (XLSX)

S1 Fig Distributions and bivariate associations between key study variables from Study 1. *Note*. C-DD = cortical marker of delay discounting; CBCL = Child Behavior Checklist (Achenbach & Rescorla, 2001).
(DOCX)

S2 Fig Distributions and bivariate associations between key study variables from Study 2. *Note*. C-DD = cortical marker of delay discounting; CBCL = Child Behavior Checklist (Achenbach & Rescorla, 2001).
(DOCX)
