# Supplementary material for: Assessing the utility of a novel cortical marker of delay discounting (C-DD) in two independent samples of early adolescents: Links with externalizing pathology
Source: PLoS One. 2023 Sep 27;18(9):e0291868. doi: 10.1371/journal.pone.0291868 (PMC10529595; doi:10.1371/journal.pone.0291868)
Supplement: S1 Appendix — (DOCX) [file pone.0291868.s001.docx]

**S1 Appendix. Calculation of C-DD originally cited by Sadeh et al (2021).**

**To calculate the Cortical Delay Discounting (C-DD) Score:** Standardize (z-score) the cortical thickness in each FreeSurfer Destrieux Atlas parcellation. Create a C-DD score by adding weighted values for each parcellation designated below. *Note*: RH = right hemisphere, LH = left hemisphere, G = gyrus, S = sulcus.

**LIST OF FREESURFER DESTRIEUX ATLAS REGIONS & ASSOCIATED WEIGHTS:**

- RH Fronto-marginal gyrus (of Wernicke) and sulcus*-0.012+
- RH Inferior occipital gyrus (O3) and sulcus*-0.039+
- RH Paracentral lobule and sulcus*-0.052+
- RH Subcentral gyrus (central operculum) and sulci*-0.046+
- RH Transverse frontopolar gyri and sulci*0.007+
- RH Anterior part of the cingulate gyrus and sulcus (ACC)*0.022+
- RH Middle-anterior part of the cingulate gyrus and sulcus (aMCC)*-0.031+
- RH Middle-posterior part of the cingulate gyrus and sulcus (pMCC)*-0.092+
- RH Posterior-dorsal part of the cingulate gyrus (dPCC)*-0.128+
- RH Posterior-ventral part of the cingulate gyrus (vPCC, isthmus of the cingulate gyrus)*-0.031+
- RH Cuneus*0.006+
- RH Opercular part of the inferior frontal gyrus*-0.005+
- RH Orbital part of the inferior frontal gyrus*-0.007+
- RH Triangular part of the inferior frontal gyrus*0.017+
- RH Middle frontal gyrus *0.005+
- RH Superior frontal gyrus*-0.023+
- RH Long insular gyrus and central sulcus of the insula*-0.047+
- RH Short insular gyri*-0.070+
- RH Middle occipital gyrus (O2, lateral occipital gyrus)*-0.049+
- RH Superior occipital gyrus (O1)*-0.002+
- RH Lateral occipito-temporal gyrus (fusiform gyrus, O4-T4)*-0.089+
- RH Lingual gyrus, lingual part of the medial occipito-temporal gyrus (O5)*-0.027+
- RH Parahippocampal gyrus, *-0.053+
- RH Orbital gyri*-0.054+
- RH Angular gyrus*-0.008+
- RH Supramarginal gyrus*-0.013+
- RH Superior parietal lobule (lateral part of P1)*0.006+
- RH Postcentral gyrus*-0.025+
- RH Precentral gyrus*-0.001+
- RH Precuneus (medial part of P1)*0.002+
- RH Straight gyrus*-0.038+
- RH Subcallosal area, subcallosal gyrus*0.036+
- RH Anterior transverse temporal gyrus (of Heschl)*-0.036+
- RH Lateral aspect of the superior temporal gyrus*-0.023+
- RH Planum polare of the superior temporal gyrus*-0.020+
- RH Planum temporale of the superior temporal gyrus*-0.013+
- RH Inferior temporal gyrus (T3)*-0.025+
- RH Middle temporal gyrus (T2)*-0.054+
- RH Horizontal ramus of the anterior segment of the lateral sulcus*0.045+
- RH Vertical ramus of the anterior segment of the lateral sulcus*0.0305+
- RH Posterior ramus of the lateral sulcus*-0.044+
- RH Occipital pole*-0.051+
- RH Temporal pole*-0.060+
- RH Calcarine sulcus*-0.011+
- RH Central sulcus (Rolando’s fissure)*-0.047+
- RH Marginal branch of the cingulate sulcus*-0.018+
- RH Anterior segment of the circular sulcus of the insula*-0.058+
- RH Inferior segment of the circular sulcus of the insula*0.017+
- RH Superior segment of the circular sulcus of the insula*-0.056+
- RH Anterior transverse collateral sulcus*-0.022+
- RH Posterior transverse collateral sulcus*-0.071+
- RH Inferior frontal sulcus*-0.017+
- RH Middle frontal sulcus*-0.001+
- RH Superior frontal sulcus*-0.034+
- RH Sulcus intermedius primus (of Jensen)*-0.013+
- RH Intraparietal sulcus and transverse parietal sulci*-0.012+
- RH Middle occipital sulcus and lunatus sulcus*-0.020+
- RH Superior occipital sulcus and transverse occipital sulcus*-0.012+
- RH Anterior occipital sulcus and preoccipital notch*-0.048+
- RH Lateral occipito-temporal sulcus*-0.014+
- RH Medial occipito-temporal sulcus and lingual sulcus*-0.058+
- RH Lateral orbital sulcus*-0.001+
- RH Medial orbital sulcus (olfactory sulcus)*0.008+
- RH Orbital sulci (H-shaped sulci)*-0.004+
- RH Parieto-occipital sulcus*-0.008+
- RH Pericallosal sulcus (S of corpus callosum)*-0.021+
- RH Postcentral sulcus*-0.001+
- RH Inferior part of the precentral sulcus*-0.037+
- RH Superior part of the precentral sulcus*-0.029+
- RH Suborbital sulcus*0.019+
- RH Subparietal sulcus*-0.023+
- RH Inferior temporal sulcus*-0.023+
- RH Superior temporal sulcus*-0.037+
- RH Transverse temporal sulcus*-0.089 +
- LH Fronto-marginal gyrus (of Wernicke) and sulcus*-0.044+
- LH Inferior occipital gyrus (O3) and sulcus*-0.058+
- LH Paracentral lobule and sulcus*-0.076+
- LH Subcentral gyrus (central operculum) and sulci*-0.033+
- LH Transverse frontopolar gyri and sulci*-0.024+
- LH Anterior part of the cingulate gyrus and sulcus (ACC)*0.030+
- LH Middle-anterior part of the cingulate gyrus and sulcus (aMCC)*0.015+
- LH Middle-posterior part of the cingulate gyrus and sulcus (pMCC)*-0.045+
- LH Posterior-dorsal part of the cingulate gyrus (dPCC)*-0.119+
- LH Posterior-ventral part of the cingulate gyrus (vPCC, isthmus of the cingulate gyrus)*0.047+
- LH Cuneus*-0.049+
- LH Opercular part of the inferior frontal gyrus*-0.000+
- LH Orbital part of the inferior frontal gyrus*-0.047+
- LH Triangular part of the inferior frontal gyrus*0.003+
- LH Middle frontal gyrus (F2)*0.007+
- LH Superior frontal gyrus (F1)*-0.0253+
- LH Long insular gyrus and central sulcus of the insula*-0.036+
- LH Short insular gyri*-0.0422+
- LH Middle occipital gyrus (O2, lateral occipital gyrus)*-0.018+
- LH Superior occipital gyrus (O1)*0.001+
- LH Lateral occipito-temporal gyrus (fusiform gyrus, O4-T4)*-0.088+
- LH Lingual gyrus, lingual part of the medial occipito-temporal gyrus (O5)*-0.024+
- LH Parahippocampal gyrus*-0.056+
- LH Orbital gyri*-0.068+
- LH Angular gyrus*0.003+
- LH Supramarginal gyrus*-0.024+
- LH Superior parietal lobule (lateral part of P1)*0.014+
- LH Postcentral gyrus*-0.030+
- LH Precentral gyrus*-0.008+
- LH Precuneus (medial part of P1)*-0.024+
- LH Straight gyrus*-0.040+
- LH Subcallosal area, subcallosal gyrus*0.048+
- LH Anterior transverse temporal gyrus (of Heschl)*-0.054+
- LH Lateral aspect of the superior temporal gyrus*-0.017+
- LH Planum polare of the superior temporal gyrus*0.003+
- LH Planum temporale of the superior temporal gyrus*0.003+
- LH Inferior temporal gyrus (T3)*-0.045+
- LH Middle temporal gyrus (T2)*-0.031+
- LH Horizontal ramus of the anterior segment of the lateral sulcus*-0.046+
- LH Vertical ramus of the anterior segment of the lateral sulcus*0.041+
- LH Posterior ramus of the lateral sulcus*-0.056+
- LH Occipital pole*-0.051+
- LH Temporal pole*-0.029+
- LH Calcarine sulcus*-0.013+
- LH Central sulcus*-0.033+
- LH Marginal branch of the cingulate sulcus*0.045+
- LH Anterior segment of the circular sulcus of the insula*-0.070+
- LH Inferior segment of the circular sulcus of the insula*0.001+
- LH Superior segment of the circular sulcus of the insula*-0.080+
- LH Anterior transverse collateral sulcus*-0.016+
- LH Posterior transverse collateral sulcus*-0.016+
- LH Inferior frontal sulcus*-0.020+
- LH Middle frontal sulcus*0.025+
- LH Superior frontal sulcus*-0.023+
- LH Sulcus intermedius primus (of Jensen)*-0.054+
- LH Intraparietal sulcus and transverse parietal sulci*0.013+
- LH Middle occipital sulcus and lunatus sulcus*-0.025+
- LH Superior occipital sulcus and transverse occipital sulcus*-0.022+
- LH Anterior occipital sulcus and preoccipital notch*0.006+
- LH Lateral occipito-temporal sulcus*-0.007+
- LH Medial occipito-temporal sulcus and lingual sulcus*-0.078+
- LH Lateral orbital sulcus*-0.019+
- LH Medial orbital sulcus (olfactory sulcus)*-0.030+
- LH Orbital sulci (H-shaped sulci)*-0.017+
- LH Parieto-occipital sulcus*-0.026+
- LH Pericallosal sulcus (S of corpus callosum)*-0.01+
- LH Postcentral sulcus*0.007+
- LH Inferior part of the precentral sulcus*-0.017+
- LH Superior part of the precentral sulcus*-0.024+
- LH Suborbital sulcus*0.030+
- LH Subparietal sulcus*-0.022+
- LH Inferior temporal sulcus*-0.046+
- LH Superior temporal sulcus*-0.023+
- LH Transverse temporal sulcus*-0.018
