# Supplementary material for: Assessing the utility of a novel cortical marker of delay discounting (C-DD) in two independent samples of early adolescents: Links with externalizing pathology
Source: PLoS One. 2023 Sep 27;18(9):e0291868. doi: 10.1371/journal.pone.0291868 (PMC10529595; doi:10.1371/journal.pone.0291868)
Supplement: S2 Fig — Note. C-DD = cortical marker of delay discounting; CBCL = Child Behavior Checklist (Achenbach & Rescorla, 2001). (DOCX) [file pone.0291868.s004.docx]

**S2 Fig.** Distributions and bivariate associations between key study variables from Study 2

*Note*. C-DD = cortical marker of delay discounting; CBCL = Child Behavior Checklist (Achenbach & Rescorla, 2001)
